# Bacterial Ghosts of *Escherichia coli* Drive Efficient Maturation of Bovine Monocyte-Derived Dendritic Cells

**DOI:** 10.1371/journal.pone.0144397

**Published:** 2015-12-15

**Authors:** Irshad Ahmed Hajam, Pervaiz Ahmad Dar, Elamurugan Appavoo, Subodh Kishore, Veerakyathappa Bhanuprakash, Kondabattula Ganesh

**Affiliations:** 1 FMD Research Center, Indian Veterinary Research Institute, Bangalore, India; 2 Division of Veterinary Microbiology and Immunology, Faculty of Veterinary Sciences and Animal Husbandry, SKUAST-Kashmir, Srinagar, India; Institut National de la Santé et de la Recherche Médicale (INSERM), FRANCE

## Abstract

Bacterial ghosts (BGs) are empty cell envelopes derived from Gram-negative bacteria. They not only represent a potential platform for development of novel vaccines but also provide a tool for efficient adjuvant and antigen delivery system. In the present study, we investigated the interaction between BGs of *Escherichia coli* (*E*. *coli*) and bovine monocyte-derived dendritic cells (MoDCs). MoDCs are highly potent antigen-presenting cells and have the potential to act as a powerful tool for manipulating the immune system. We generated bovine MoDCs *in vitro* from blood monocytes using *E*. *coli* expressed bovine GM-CSF and IL-4 cytokines. These MoDCs displayed typical morphology and functions similar to DCs. We further investigated the *E*. *coli* BGs to induce maturation of bovine MoDCs in comparison to *E*. *coli* lipopolysaccharide (LPS). We observed the maturation marker molecules such as MHC-II, CD80 and CD86 were induced early and at higher levels in BG stimulated MoDCs as compared to the LPS stimulated MoDCs. BG mediated stimulation induced significantly higher levels of cytokine expression in bovine MoDCs than LPS. Both pro-inflammatory (IL-12 and TNF-α) and anti-inflammatory (IL-10) cytokines were induced in MoDCs after BGs stimulation. We further analysed the effects of BGs on the bovine MoDCs in an allogenic mixed lymphocyte reaction (MLR). We found the BG-treated bovine MoDCs had significantly (p<0.05) higher capacity to stimulate allogenic T cell proliferation in MLR as compared to the LPS. Taken together, these findings demonstrate the *E*. *coli* BGs induce a strong activation and maturation of bovine MoDCs.

## Introduction

The bacterial ghosts (BGs) are empty cell envelopes of Gram-negative bacteria produced by the controlled expression of lysis gene *E* of bacteriophage PhiX174 [1, 2]. The gene *E* codes for a very small protein which is 91 amino acids in length that contain hydrophobic moieties within its N-terminal region. When expressed, protein E oligomerizes into a transmembrane tunnel structures in the cell envelope of host bacteria [2, 3, 4]. The E specific tunnel structure are ~ 40–200 nm in diameter and usually get localized at the membrane adhesion sites within the host cell, spanning the inner and outer membrane through which cytoplasmic contents including DNA are expelled out, leaving behind empty cell envelopes known as BGs [4, 5]. Electron microscopy analyses have revealed that empty bacterial envelopes maintain the cellular morphology similar to native bacteria where all cell surface structures including outer membrane proteins, adhesins, LPS and peptidoglycan layer are preserved [6]. In addition, the foreign antigens have been loaded inside the cytoplasmic lumen or expressed both on the surface and in the periplasmic space of BGs [6, 7]. These remarkable properties make BGs an attractive tool for vaccine development and antigen delivery system for both humans and animals. Previous studies demonstrate BGs mediate active immunization against their own envelope structures or when used as antigen delivery vector [7, 8]. Other studies found it safe and potent adjuvant which is capable of polarizing the immune response toward Th1 or Th2 depending upon the presentation of antigen [8]. The DCs are unique antigen presenting cells (APCs) with ability to prime effective immune responses and permit the establishment of immunological memory [9, 10]. The DCs represent a heterogeneous effector population exhibiting functions including regulation of T cell responses, differentiation of Th1, Th2, or Treg cells, and regulation of humoral immune responses [11, 12]. Immature DCs encounter the foreign antigens in the periphery, capture and process them, and then migrate to secondary lymphoid organs. The interaction of foreign antigens with DCs in the periphery delivers the maturation signals to DCs through up-regulation of MHC and co-stimulatory molecules, thereby presenting foreign antigens to inexperienced T cells in the correct configuration necessary for the elicitation of potent adaptive immunity [13, 14]. There is a discrepancy in the literature regarding the function of mature DCs. Some investigators report that mature DCs not only initiate T cell activation and proliferation but also have a role in the induction and maintenance of tolerance [15, 16]. Others report that tolerance induction is due to an inability of immature DCs to deliver proper costimulatory signals [17]. In either case, the role of mature DC needs to be further analysed to better understand the initiation of immune responses and tolerance induction. Nevertheless very little is known about the interaction of BGs with dendritic cells (DCs) and their impact on the maturation of DCs or induction of Th1/Th2 cytokines.

Here we report generation of bovine MoDCs from blood monocytes using recombinant bovine Granulocyte-macrophage colony-stimulating factor (GM-CSF) and Interleukin 4 (IL-4). Our data indicate differentiation of monocyte into MoDC with morphological and phenotypic features require 4–5 days. We also observed the BGs drive efficient maturation signals to DCs which were characterized by upregulation of costimulatory molecules and higher cytokine induction. Overall, the BGs enhanced stimulatory effect of DCs in a mixed lymphocyte reaction (MLR) further strengthens their potential to be used as an adjuvant and/or candidate vaccines in mammals.

## Materials and Methods

### Ethics Statement

This study was approved by the Committee for the Purpose of Control and Supervision of Experiment on Animals (CPCSEA), the Ministry of Environment and Forests, Government of India (#460/15/ab/CPCSEA). All the animal protocols were reviewed and approved by the Institute Animal Ethics Committee (IAEC) of the FMD Research Center, Bangalore, India.

### Preparation of bacterial ghosts

BGs of *E*. *coli* were produced by the controlled expression of lysis gene *E* of the bacteriophage PhiX174 [1]. The gene *E* was amplified from PhiX174 RFI DNA (#DO031, Fermantas, USA) using in-house designed gene-specific primers: 5′-gcgccatggtacgctggactttgtgggat-3′ and 5′-cgcctcgagttactccttccgcacgtaatt-3′ (S1A Fig). The PCR amplified product was cloned in pET28a vector (Novagen, San Diego, USA) using *Nco1* and *Xho1* restriction enzyme sites. The positive clones were confirmed by colony PCR and nucleotide sequence analysis. The recombinant plasmid pET28a-*E* was transformed into *E*. *coli* BL21 (DE3) strain (Novagen, USA) for expression of gene *E*. For preparation of BGs, 25 ml of Luria-Bertani (LB) broth containing Kanamycin (100 μg/ml) was inoculated with a single fresh colony of *E*. *coli* BL21 harbouring lysis plasmids. Bacteria were grown in culture medium (pH∼7.2) with agitation to a mid-log phase (OD600nm = 0.25). Expression of the lysis gene *E* was induced by the addition of 1 mM isopropyl β‑D-1-thiogalactopyranoside (IPTG) as described earlier [18]. The growth and lysis of the bacteria were monitored by measuring the optical density (OD_600nm_) and by determining the colony forming units (CFU/ml) on nutrient agar plates as described earlier [19]. The lysis of the bacteria was allowed for 5 hr and any remaining intact bacteria were killed by the addition of minimal inhibitory concentration of H_2_0_2_ (5.82 μl/ml) in phosphate buffer saline (PBS). The presence of any viable bacteria was tested by inoculation of the culture onto a nutrient agar plate and incubation at 37°C for 7 days. BG preparations were analysed for any genomic DNA contamination as described earlier [19]. The BGs were recovered by centrifugation at 10,000 x g for 15 minutes and washed thrice with PBS. Finally, the pellet was resuspended in PBS (pH = 7.2) and stored at -20°C till further use.

### Characterization of bacterial ghosts

BGs were characterized for their surface morphology by field emission scanning electron microscopy (FE-SEM) and the internal structures by transmission electron microscopy (TEM) as described earlier [20]. Briefly, BGs were recovered from 10 ml of culture and the pellet was resuspended in 300 μl of PBS. Approximately 10 μl volume of the suspension was placed on a silicon wafer and dried at 37°C. The air dried sample was fixed in 2.5% glutaraldehyde in PBS (pH 7.4), followed by serial dehydration in ethanol. Samples were dried to a critical point and coated with a gold-palladium alloy for subsequent observation by SEM (FEI, Nova NanoSem 600, USA) operated at an accelerating voltage of 5 kV. To prepare negatively-stained TEM specimens, 15 μl of *E*. *coli* ghost suspension was placed on carbon-coated copper grids and permitted to stand for 2 min to allow films to form. The extra solution was washed off using blotting paper and stained with uranyl acetate. The grids were allowed to dry for 3 hr and subsequently analysed by TEM (FEI Tecnai^™^, USA) operated at an accelerating voltage of 120 kV.

### Expression of bovine GM-CSF and IL-4

Cytokine genes were amplified from cDNA of bovine peripheral blood mononuclear cells (PBMCs) using in-house designed gene-specific primers; 3′-atccatggcacctactcgcccacccaac-5′ / 3′-gcctcgagcttctgggctggttccca-5′ for GM-CSF and 3′-cgccatgggtcacaagtgtgatattacctta-5′ / 3′-gcgctcgagacacttggagtatttctc-5′ for IL-4 (S2A Fig). The cytokine genes were cloned into pET28a expression vector (S2B Fig) and protein expression was performed as described earlier [21].The recombinant proteins were expressed with authentic N-terminals which is a prerequisite for their biological activity. The proteins were purified under denaturing conditions using Ni-NTA cartridge (Qiagen, Hilden, Germany) as per the manufacturer’s instructions. The purified proteins were refolded and dialyzed against PBS (pH 7.4) at 4° and protein concentration was determined by a Bradford assay [22]. The biological activity of rGM-CSF and rIL-4 were determined by proliferation assays using TF-1 cells [23].

### Generation and stimulation of bovine monocyte-derived dendritic cells

Blood was collected from Hallikar breed of cattle (*Bos indicus*) and PBMCs were isolated using Histopaque-1077 (Sigma–Aldrich, USA) as described previously [24]. The PBMCs were resuspended in RPMI-1640 media (with 1% FCS) and allowed to adhere to culture plates for 3 hr. The non-adherent cells were washed off and the adherent cells were further cultured in RPMI-1640 media supplemented with 10% FCS, streptomycin (100μg/ml), 50 mM 2-mercaptoethanol and cytokines (40 ng/ml of GM-CSF and 20 ng/ml of IL-4). The culture media was replaced after every 2 days supplemented with the same concentration of the cytokines. MoDCs were harvested after day 5 for further experiments. For stimulation experiments approximately 1 x 10^6^/ml MoDCs were cultured in 6 well plates in complete RPMI-1640 media and treated with either BGs (40 particles/cell) or LPS (500 ng/ml) for 0, 6 and 12 hrs.

### Real-time PCR assay

The MoDC cultures stimulated with either BGs or LPS were harvested and total RNA was isolated by RNeasy Mini kit (Qiagen, Hilden, Germany) as described by the manufacturer. The concentration and purity of RNA were checked by spectroscopy using a NanoDrop Spectrophotometer (Thermo Scientific, Pittsburgh PA, United States). RNA with A280/A260 ratio in the range of 1.8–2.0 was considered pure and used for the cDNA synthesis. The cDNA was prepared from 1.0 μg of RNA using the SuperScript^™^ III Reverse Transcriptase kit (Invitrogen, San Diego, California, USA) as described earlier [24] and stored at -20°C until further use. A real-time PCR assay (qRT-PCR) for gene expression studies (Table 1) was performed with the ABI applied biosystems using Power SYBR Green PCR Master Mix (#4367659, Applied Biosystems, USA) as described previously [24]. The relative amounts of cytokine mRNA present (normalized with GAPDH) were determined by 2^-ΔΔCT^ method [24].

**Table 1:** List of primers used in this study. The expected size of PCR products and the efficiency of the primers obtained by the qRT-PCR are given.

Target gene	Primer sequence (5’-3’)	Product size	Primer efficiency	Accession #/ Reference
IL-10	TGTTGACCCAGTCTCTGCTG	154 bp	1.99	U00799
	AGCTTCTCCCCCAGTGAGTT			
IL-12	GAGGCCTGTTTACCACTGGA	141 bp	1.92	[47]
	CTCATAGATACTTCTAAGGCACAG			
TNF-α	CCATCAACAGCCCTCTGGTT	138 bp	2.0	[48]
	CCATGAGGGCATTGGCATAC			
GAPDH	GGCGTGAACCACGAGAAGTATAA	194 bp	1.98	[47]
	CCCTCCACGATGCCAAAGT			
TLR2	ACGACGCCTTTGTGTCCTAC	192 bp	1.95	[49]
	CCGAAAGCACAAAGATGGTT			
TLR4	ACTGACGGGAAACCCTATCC	208 bp	1.98	[49]
	CAGGTTGGGAAGGTCAGAAA			
TLR5	AAAACCACATCGCCAACATC	191 bp	1.98	[49]
	CATCAGATGGAACTGGGACA			
TLR6	CAAAGTGGGGAACAATCCAT	206 bp	1.95	[49]
	CCACAATGGTGACGATCAGC			
MHCII	CGAGTGGAACCTACAGTGAC	95 bp	2.0	NM_001034668.3
	CTGGGATAGAAATCCGTCAC			
CD14	CAGCTCTCTGTCCGAACAAG	193 bp	2.0	[30]
	CCTTAGTGCACTGGGCCAG			
CD40	TGTTGTCTGCGGTTTCCAGA	118 bp	1.9	NM_001105611.2
	CCGCTTCTTGGTTATGTTCCTG			
CD80	ACCACCCAAGCGCCCATG	210 bp	2.0	[30]
	AGGCAGGATGGCCAGCAC			
CD86	TGTGCCCTGCAACTTGAGCCA	190 bp	2.0	[30]
	AGAGGGGCCAGGCTGCTTCT			

### Mixed leukocyte reaction

The MoDCs or monocytes were cultured in triplicate in U-bottom 96-well culture plates and mixed with 1.1 x 10^5^ allogenic T cells/well containing media alone or media supplemented with either BGs (40 particles/cell), ConA (0.5 ng/well) or LPS (100 ng/well) from *E*. *coli* (Sigma Aldrich, USA). For this purpose, T cells were purified from PBMCs over a nylon wool column as described elsewhere [25]. The proliferation of the T cells was measured using MTT based assay as described earlier [26]. The T cell proliferative responses for each culture condition were expressed as a stimulation index (SI), where SI was calculated by measuring absorbance of T cells and myeloid cells divided by absorbance of T cells in a medium.

### Statistical Analysis

The statistical analysis was performed using GraphPad prism 5.00 program (San Diego, CA, USA). The data were analysed by two-tailed unpaired student’s t-test to compare the data from gene expression studies performed on sorted samples. A one-way ANOVA with Tukey`s multiple comparison test was used between different groups. The data are represented as mean ± standard deviation and *p* values < 0.05 were considered statistically significant.

## Results

### Production and characterization of bacterial ghosts of *E*. *coli*

**Fig 1:**
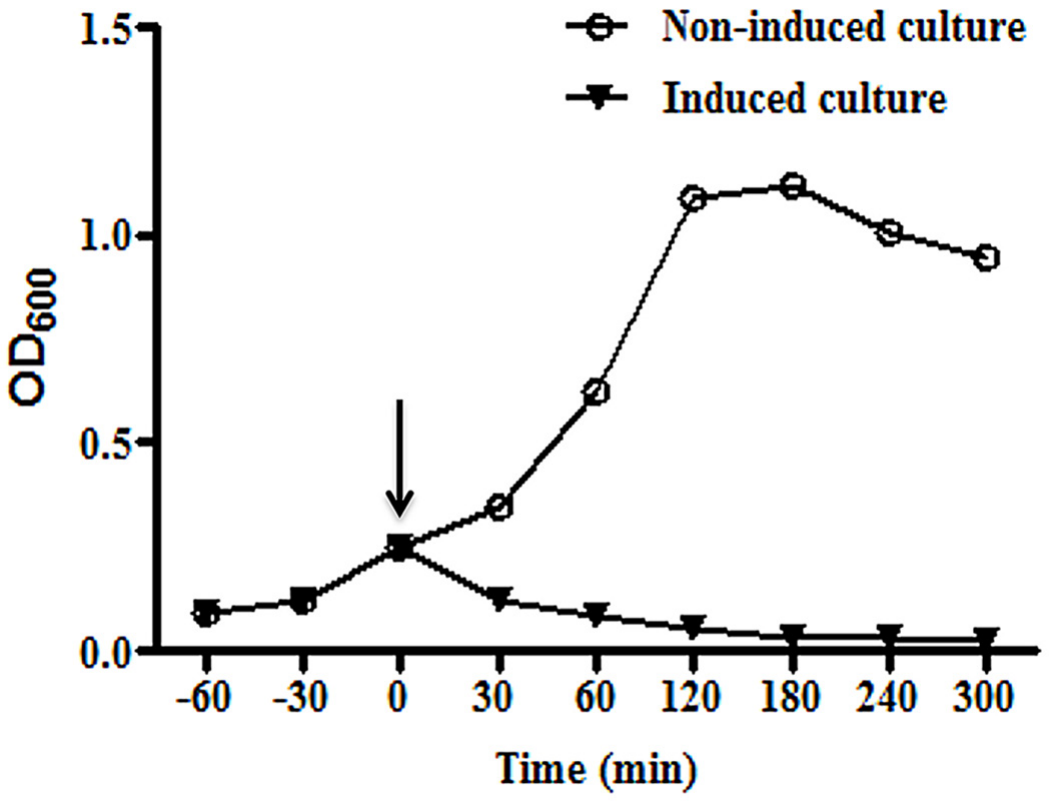
Growth kinetics of *E*.*coli* ghosts harbouring pET28a-*E* lysis plasmid. The transformed bacteria with gene *E* of bacteriophage PhiX174 was grown at 37°C until mid-log phase (OD600 = 0.25). The culture was split and one part was induced by the addition of 1 mM IPTG whereas the other part served as a non-induced control. The growth was monitored by measuring the optical density (OD_600nm_) of culture at various time points. After induction (indicated by arrow), BGs showed rapid decrease in OD indicative of lysis whereas the uninduced culture showed growth of bacteria as indicated by an increase in OD.

The gene *E* was cloned and expressed in *E*. *coli* BL21 (DE3) strain with authentic N and C-terminals which is a prerequisite for the lysis activity. The onset of lysis started as early as half an hour post induction as indicated by a decrease in OD_600nm_ (Fig 1). We observed fresh bacterial cultures exhibit efficient lysis as compared to the relatively old cultures (data not given). Generally the *E*. *coli* cultures with OD values above 0.4 showed inefficient lysis process while cultures with OD values between 0.2–0.3 resulted in highly efficient BG production. This finding implies that gene *E* mediated lysis is dependent on the active phase of bacterial growth as reported earlier [4, 27]. The results of four replicate experiments showed that the lysis process (~5 hr) caused almost 99.97% decrease in live bacteria. The data also indicated that killing of *E*. *coli* was never absolute as reported previously [19]. In order to kill any residual live bacteria, we added H_2_O_2_ to the cultures and growth of any viable bacteria was analysed by spreading 100 μl of BG culture on a nutrient agar plate. We observed no growth on the agar plate after 7 days of incubation at 37°C. Moreover, agarose gel electrophoresis revealed BGs free of any genomic DNA contamination, indicating complete inactivation of DNA (S1B Fig). The addition of H_2_O_2_ to the culture is advantageous over the use of antibiotics as it completely inactivates any remaining genomic DNA [28].

**Fig 2:**
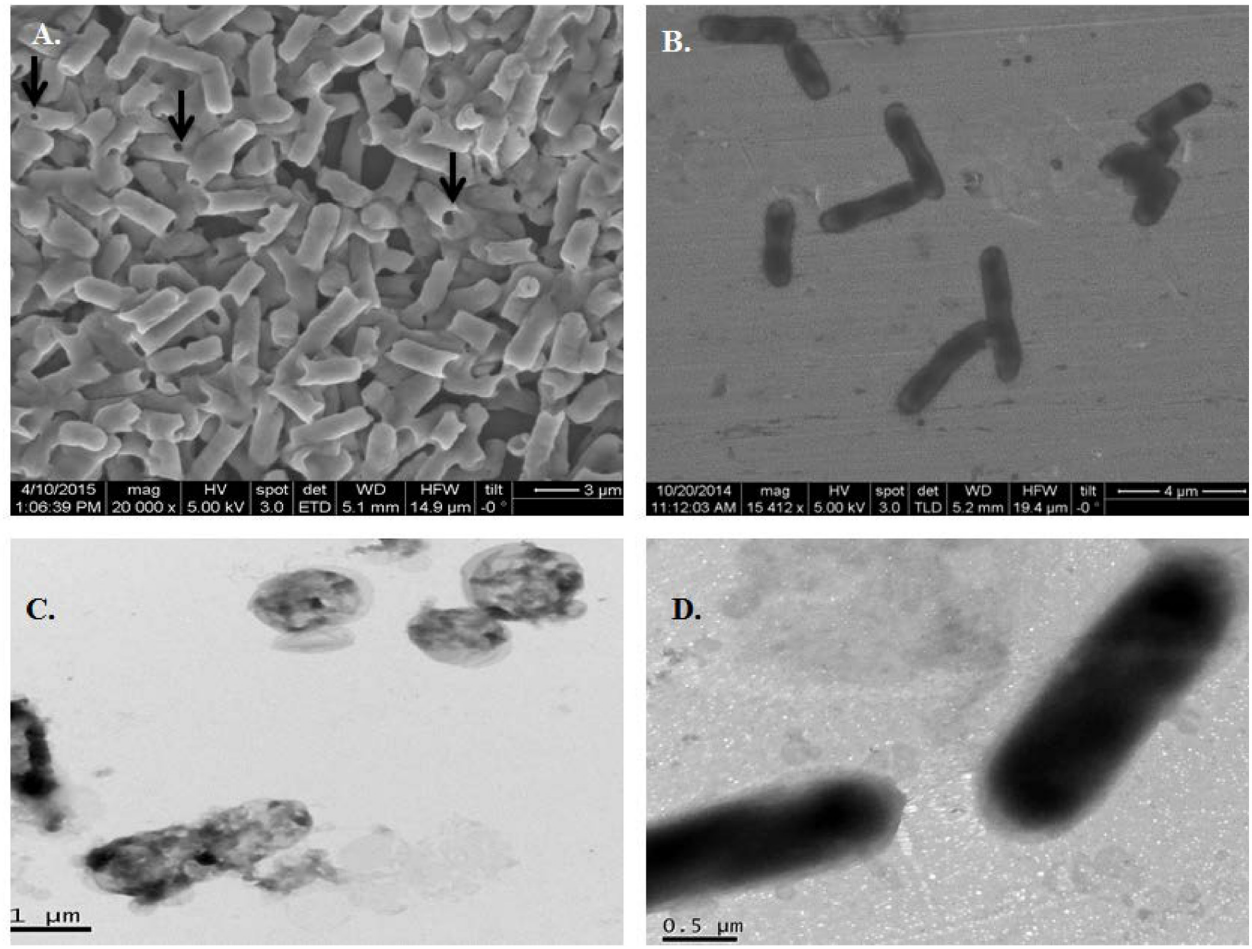
Characterization of *E*. *coli* ghosts by electron microscopy. (A) Field emission scanning electron micrograph (FE-SEM) of protein E lysed *E*. *coli* ghosts showing transmembrane tunnels, indicated by arrow heads. (B) FESEM of intact *E*. *coli* before lysis. (C) Transmission electron micrograph (TEM) of *E*. *coli* ghosts with a loss of cytoplasmic contents but intact cellular morphology. (D) TEM of intact *E*. *coli* prior to gene *E* induction.

Electron microscopic analysis of *E*. *coli* ghosts revealed no gross alterations in the cellular morphology of BGs compared to the unlysed cells, except the presence of transmembrane tunnels (Fig 2A and 2B). The SEM studies revealed transmembrane pores in BGs ranging in size between 50 and 210 nm. The TEM analysis confirmed BGs were devoid of any internal structures as compared to the unlysed cells that contained cytoplasmic contents (Fig 2C and 2D).

### Morphological and phenotypic characteristics of monocytes vs. MoDCs

**Fig 3:**
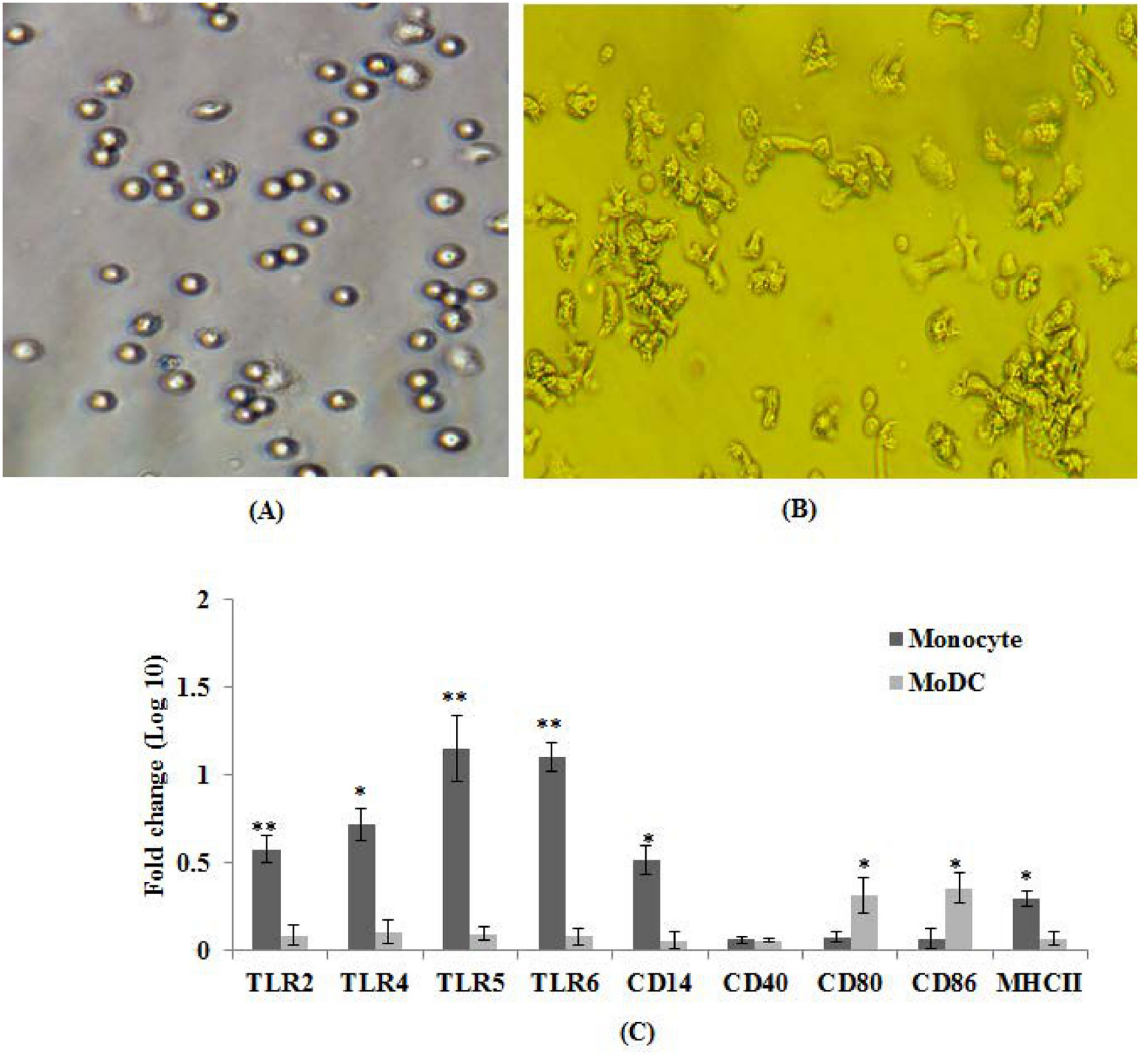
Morphological and phenotypic characterization of immature MoDCs. (A) Light microscopy (20X) of freshly isolated monocyte. (B) Light microscopy (20X) of 4^th^ day culture of presumably immature MoDCs showing clusters of veiled cells with pseudopodia. (C) Freshly isolated monocytes and 5 day culture of MoDCs were analysed for phenotypic changes by evaluating mRNA expression levels of various TLRs and costimulatory genes by qRT-PCR assay. Results are expressed as fold change (log10) in mRNA transcription of monocytes and MoDCs. Data presented are mean ± standard deviation of values obtained from three independent experiments involving two cattle of Hallikar breed. *p< 0.05.**p< 0.01.

We used *E*. *coli* expressed GM-CSF and IL-4 cytokines for differentiating adherent monocytes into MoDCs. The SDS-PAGE analysis revealed the purity of both the recombinant cytokines used for MoDC differentiation studies well above 95% (S2C Fig). The recombinant cytokines were biologically active and showed best results at concentrations of 40 and 20 ng / ml, respectively. At these concentrations, 90–95% of the monocytes differentiated into DCs as observed under microscopy. However, complete differentiation of monocytes into veiled cells with pseudopodia required 4–5 days (Fig 3A and 3B) much like human system. The higher concentration of cytokines resulted in the monocytes differentiating into long-stretched fibroblastic cells known to be present in both equine and human systems [29].

We analysed monocytes and MoDCs by qRT-PCR to investigate any difference in the expression of innate immune receptors (TLR), CD14 and co-stimulatory molecules. Monocytes expressed significantly (p<0.05) higher levels of TLRs than MoDCs (Fig 3C), suggesting that differentiation of monocytes into DCs resulted in decreased expression of TLRs [30]. As expected the MoDCs shed CD14 gene expression and the mRNA levels were significantly (p<0.05) lower than monocytes. The MoDCs expressed significantly (p<0.05) higher levels of costimulatory molecules CD80/CD86 than monocytes, but no significant difference was observed in CD40 gene expression. The MoDCs also had relatively lower levels of MHC-II than monocytes and this finding was in agreement with the earlier report [31].

### Bacterial ghosts enhance costimulatory molecules on the surface of MoDCs

**Fig 4:**
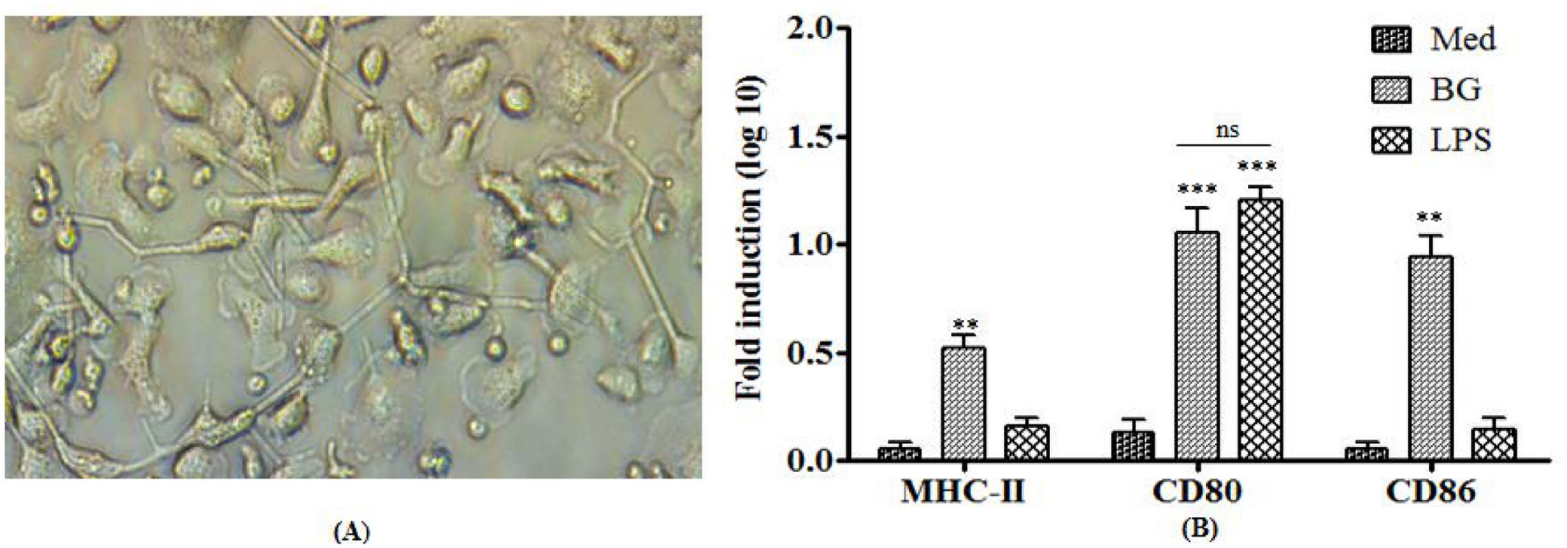
Morphological and phenotypical features of mature bovine MoDCs. (A) Light microscopy (20X) of MoDCs after stimulation with BGs demonstrated large dendrite like pseudopodia (B) qRT-PCR analysis of MoDCs after stimulation with either BGs or LPS or treated with media (Med). Results are expressed as fold induction (log10) in mRNA transcription after BGs or LPS stimulation compared to the media treated (non-stimulated) cells. Gene expressions were normalized to GAPDH and mRNA levels at 0 h were used as calibrator. Data presented are mean ± standard deviation of three independent experiments involving two cattle of Hallikar breed. **p < 0.01, ***p<0.001, ns = non-significant.

The DCs remain immature cells without any external stimuli and may be activated by microbial components. In the present study, we investigated the effect of BGs and LPS on the maturation state of DCs in the context of morphological changes and costimulatory molecules. After stimulation, both BGs and LPS induced morphological changes as indicated by the presence of long dendrites in stimulated cells as compared to immature MoDCs (Fig 4A). The unstimulated MoDCs expressed low levels of MHC-II, CD80, and CD86 costimulatory molecules. The BG stimulation significantly (p<0.01) enhanced MHC-II, CD80, and CD86 expression levels by 6 hrs (Fig 4B). In contrast, the LPS stimulation upregulated CD80 to significantly higher levels (p<0.001) but had no effect on MHC-II and CD86 molecules during the 6 hrs experiment. These findings indicate that BGs deliver efficient and earlier maturation signals to immature MoDCs, a prerequisite for initiating a subsequent phase of protective immunity dominated by pathogen-specific T and B cells.

### Bacterial ghosts induce higher cytokine responses in MoDCs

**Fig 5:**
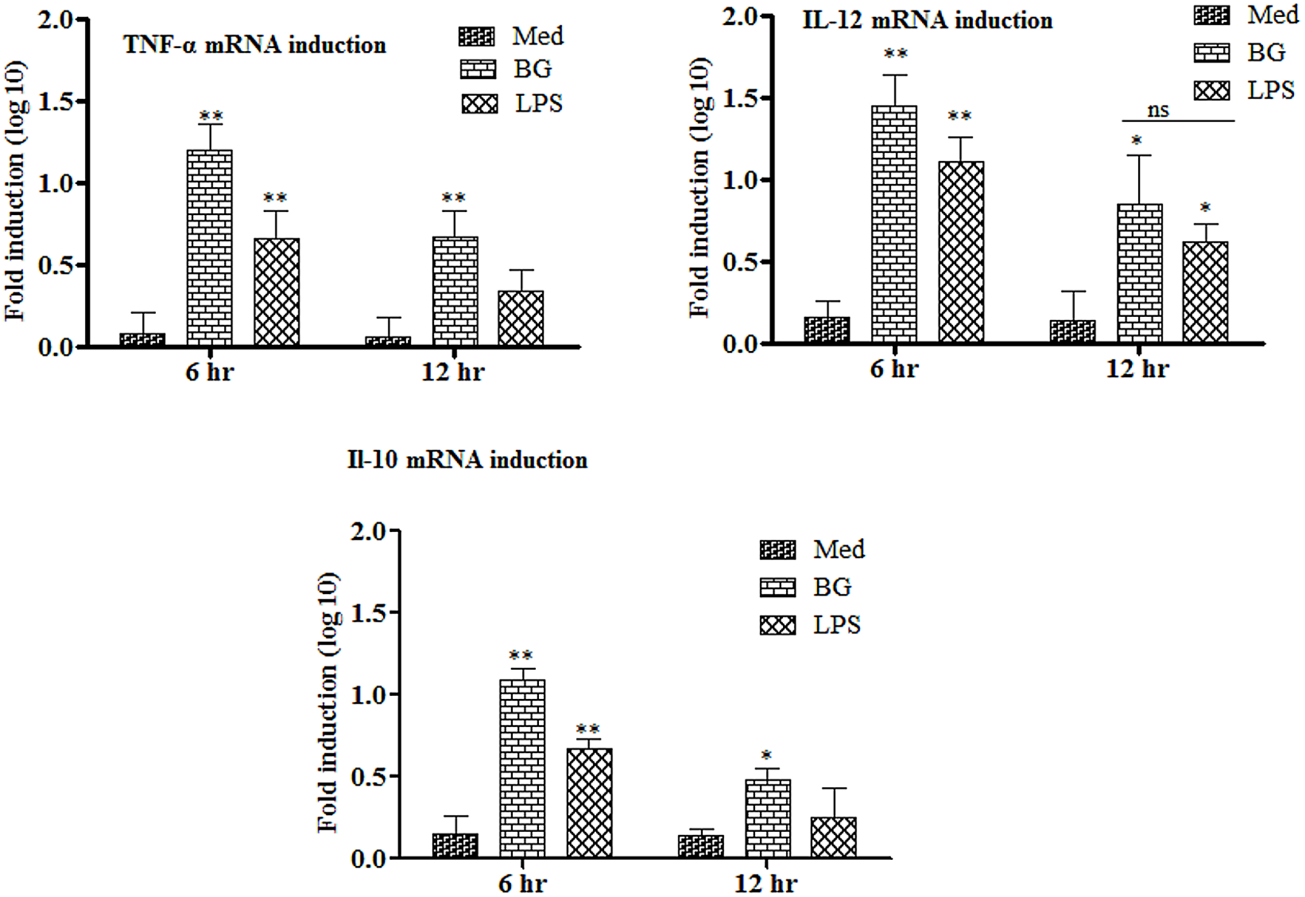
Kinetics of cytokine mRNA transcription from MoDCs treated with either BGs or LPS. RNA was extracted and gene transcription was quantified by qRT-PCR at 0, 6 and 12 h. Results are expressed as fold induction (log10) of cytokine mRNA transcription by BGs or LPS stimulated cells compared to the media treated (Med) cells. GAPDH was used as an internal control and mRNA levels at 0 h was used as calibrator. Histograms represent mean cytokine levels and bars represent standard deviation of three independent experiments. *p < 0.05, **p<0.01, ns = non-significant.

Here we analysed the ability of MoDCs to respond to external stimuli in the context of the kinetics of cytokine mRNA induction. The pattern and levels of cytokine mRNA induction varied depending on both the cytokine being examined and the stimuli being used. In our hands, both BG and LPS treatments induced cytokine expression in MoDCs. However, BG induced significantly (p<0.05) higher levels of cytokine expressions in MoDCs than the LPS (Fig 5).

The BGs induced significantly higher levels (p<0.01) of IL-12 in MoDCs at 6 hrs post stimulation that remained significantly higher (p<0.05) till 12 hrs of treatment. In contrast, the LPS induced relatively low levels of IL-12 but showed similar kinetics. Both BGs and LPS enhanced the levels of IL-10 in MoDCs. The IL-10 levels were significantly (p<0.01) higher at 6 hrs post stimulation which decreased significantly (p<0.05) at 12 hr in the case of BG and non-significant in the case of LPS. TNF-α, a proinflammatory cytokine, was also induced and showed kinetics similar to those of IL-10. Our results are in accord with the previously published report that showed DCs maturation using TLR agonists induced effective cytokine production at 8 hrs post stimulation [32].

### Bacterial ghosts increase stimulatory effect of MoDCs in allogenic MLR

**Fig 6:**
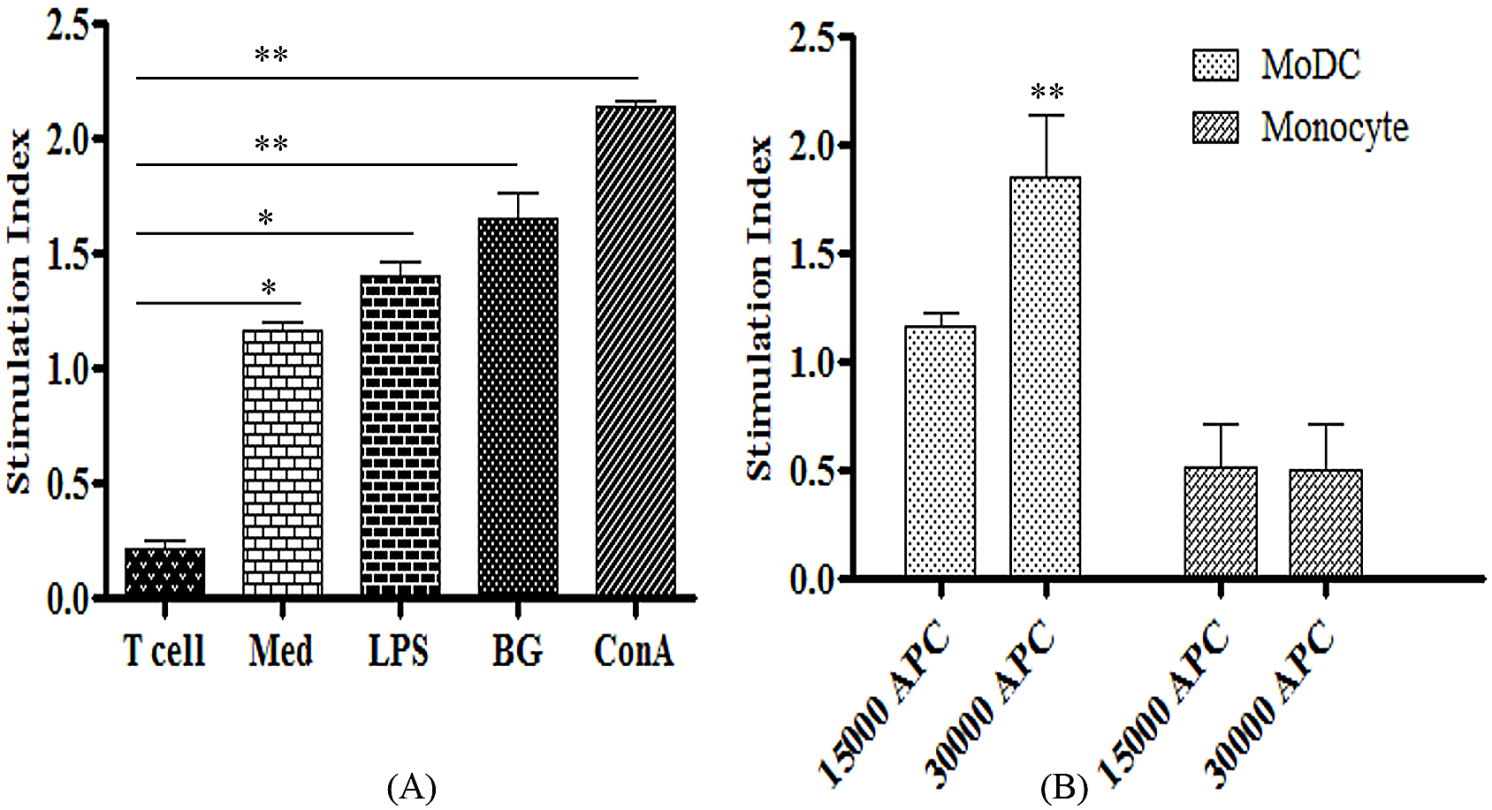
Antigen presenting capacity of MoDCs determined by mixed lymphocyte reaction. (A) Bovine MoDCs (1.5 x 10^4^) were cultured with allogenic T cells (1.1 x 10^5^) and treated either with ConA, BGs, LPS or media (Med). The T cells alone stimulated either with LPS or media were kept as controls. (B) MoDCs were cultured with allogenic T cells (1.1 x 10^5^) of a different breed in a graded manner. The cultures were maintained for three days and subsequently, T cell proliferation was measured by the uptake of MTT dye and expressed as stimulation index. Data presented are mean ± standard deviation of values for MoDCs and allogenic T cells isolated from three animals. *p<0.05.**p<0.01.

The hallmark of DCs is their ability to present antigens and stimulate naïve T cells *in vivo*, unlike monocytes and B cells. This remarkable property can be best analysed *in vitro* by the primary MLR as neither B cells nor macrophages stimulate T cells *in vitro*. We tested MoDCs in MLR with allogenic T cells. In the absence of external stimuli, the MoDCs activated allogenic T cells significantly (p<0.05) in a dose-dependent manner (Fig 6A and 6B). To our understanding, the stimulatory effect of MoDCs on T-cells had improved when stimulated by either the BGs or LPS. However, the BGs displayed significantly increased (p<0.01) stimulatory effect on MoDCs by 1.42 and 1.17 fold higher compared to media and LPS, respectively. In addition, the LPS increased stimulatory effects of MoDCs to 1.16 fold higher than media. The ConA, a polyclonal T cell activator, showed the highest (p<0.01) T cell proliferation responses (Fig 6A). These findings strongly support the notion that DCs efficiently present foreign antigens to naïve T cells and have a clear role in the initiation of adaptive immune responses.

## Discussion

The DCs are considered most potent APCs and are essential in the understanding of regulatory mechanisms of immune systems. They are the only cells to prime naïve T cells and initiate primary immune responses [10]. The biology of DCs is very intriguing with distinct subpopulations expressing varying phenotypic markers and functional activities in almost all mammalian species [12, 13, 30, 33]. There is an increasing interest in understanding DC biology as they have the ability to modulate the immune response either towards specific effector functions or tolerance. In the present study, we explored the interaction of bovine MoDCs with *E*. *coli* BGs and showed that the BGs drive efficient maturation and activation of the bovine MoDCs.

The differentiation of DCs from monocytes has been shown earlier to be dependent on species specific GM-CSF and IL-4 [29, 31]. The isolation of bovine myeloid DCs (mDCs) directly from the blood is inefficient due to their very low occurrence, inefficient isolation protocols and lack of lineage specific markers [30]. Moreover, isolation of non-adherent cells from peripheral blood and subsequent enrichment of DCs using metrizamide gradient usually activates these cells for accessory activities [29]. Furthermore, these cells succumb to death rapidly without GM-CSF *in vitro*. Therefore, the direct isolation methods seem far less optimal for obtaining DCs in large numbers and evaluating them *in vitro*. In the present study, we isolated bovine monocytes by the adherent method and differentiated the monocytes into MoDCs using species specific GM-CSF and IL-4 which have been reported in number of studies [29, 31, 34]. The recombinant cytokines were functionally active in generating MoDCs which were quite distinct from monocytes and macrophages both morphologically and phenotypically. Morphologically, the bovine MoDCs were similar to DCs of humans and equines [29, 34]. However in contrast to 6–7 days requirement to generate equine MoDCs, the bovine monocytes took only 4–5 days to differentiate very similar to the human system [34]. Expression analysis of MoDCs revealed that these cells were CD14^lo^, CD86^hi^ and CD86^hi^ which resemble human MoDCs and cattle blood-derived DCs [30, 33, 35, 36]. However, these cells expressed relatively low levels of MHC-II and displayed TLR gene expression distinct than monocytes. In the present study down regulation of CD14 and upregulation of costimulatory molecules strongly supports the differentiation of MoDCs, similar finding have been reported previously [30, 29, 31]. These data suggest that monocytes were able to fully differentiate into MoDCs using in-house *E*. *coli* expressed GM-CSF and IL-4.

All the adjuvants including TLR agonists work in part by inducing DC activation/maturation and their recruitment to T cell areas of lymph nodes [13]. The DCs activation and maturation are indicated by the upregulation of costimulatory molecules and secretion of cytokines like IL-12, TNF-α and IL-18 [37, 38]. In the present study, we investigated the potential of BGs to induce activation and maturation of bovine MoDCs. We noticed both BGs and LPS induce morphological changes consistently but variations in the phenotypic changes. The BGs markedly upregulate all costimulatory molecules and induce earlier maturation in contrast to LPS which showed upregulation of CD80 only. Previous study showed *Salmonella* LPS and BGs of *Mannheimia haemolytica* induces similar level of activation of mouse MoDCs [39]. This variation could be due to species difference as has been reported with equines DCs [29], nature of BGs [39] or variable response to LPS by different DC subsets [33, 38]. Despite the acquisition of partial maturation state by LPS stimulation, it has to be stressed that MoDCs were functionally active in MLRs, probably receiving some final maturation signals through CD40-CD40L interaction.

BGs induce significantly higher levels of IL-12 followed by TNF-α and IL-10 in comparison to LPS. IL-12 is a heterodimeric cytokine crucial for polarizing immune responses towards the Th1 type. Effective DC maturation and its functioning depend not only on the sufficient expression of antigen and costimulatory molecules but also on the secretion of interleukin IL-12 [40]. The IL-12 is used to access the maturation state and serves as a functional marker of DCs [40]. The IL-12 is the main cytokine driving the stimulation of NK cells and Th1 cells, thereby causing the elicitation of protective immunity against infectious and non-infectious diseases [41]. The DCs not only stimulate T cells but have the potential to activate naïve and memory B lymphocytes [42]. The interaction of DCs with B cells plays a very important role in the generation of humoral responses and occurs through the expression of CD40L and cytokines [43]. The present study illustrates that BGs significantly enhance IL-10 and TNF-α levels. This upregulation is key because IL-10 and TNF-α are the main cytokines involved in immunoglobulin class switching, an important phenomenon in humoral immunity. The IL-10 has a clear role in IgA production in cattle [44] and in humans promotes class switching to IgG3, IgG1 and IgA [45]. The TNF-α is also important for stimulating Th2 cells to promote the class switching in B cells [46]. These findings clearly indicate that BGs deliver efficient maturation signals to DCs necessary for the subsequent activation of T cells and prevention of tolerance to infectious non-self antigens.

In conclusion, this study indicates the *E*. *coli* BGs deliver efficient and earlier maturation signals to DCs as compared to LPS. Importantly, BG stimulation results in both pro-inflammatory (TNF-α and IL-12) and anti-inflammatory (IL-10) cytokine responses in bovine MoDCs. This study also indicates that BGs have the potential to act as adjuvants and/or candidate vaccines through improving maturation and functioning of DCs. The observations made in the study need to be followed-up by further animal studies to confirm the efficiency of BGs as an adjuvant for vaccines.

## Supporting Information

S1 Fig PCR amplification of gene E of bacteriophage PhiX174 and analysis of genomic DNA content of BGs. (A) Gene E was amplified from PhiX174 RFI DNA using gene specific primers. Lanes; 1, represent gene *E* (273 bp); 2, negative control; M, represent 1 kb marker (#SM0313, ThermoScientific, United States). (B) BGs were analysed for contamination of genomic DNA. To end this, samples were taken after induction at various time points. 1 ml of bacterial culture was centrifuged and subsequently, supernatant and pellet was analysed in 0.5% agarose gel electrophoresis. Lanes; 1, represent 0 hr culture showing genomic DNA at the well; 2, supernatant of bacterial culture post 1 hr induction showing degradation of genomic DNA; 3, pellet of bacterial culture post 4 hr induction free of genomic DNA; 4, supernatant of bacterial culture post 4 hr induction showing complete inactivation of genomic DNA; M, represent 1 kb marker.
(TIF)

S2 Fig PCR amplification and SDS-PAGE analysis of GM-CSF and IL-4recombinant proteins. (A) GM-CSF (lane 1) and IL-4 (lane 2) were amplified using gene specific primers from bovine cDNA. Lane M represent 1 kb marker (#SM0313, ThermoScientific, United States). (B) Confirmation of GM-CSF or IL-4 gene in pET28a vector by colony PCR. Colony PCR showing amplification of 378 bp of GM-CSF (lanes 1–3) and 333 bp of IL-4 (lanes 4–5). (C) SDS–PAGE analysis of purified products of GM-CSF and IL-4. GM-CSF or IL-4 recombinant plasmid was transformed into *E*. *coli* BL21 (DE3) host strain for expression. The expressed proteins were purified by Ni-NTA cartridge as described in material methods. Lanes; 1–4, represent GM-CSF; 5–6, represent IL-4; M, molecular weight marker (#PG500-0500PI, ThermoScientific, US).
(TIF)
