# Supplementary figures and images for: Bacterial Ghosts of Escherichia coli Drive Efficient Maturation of Bovine Monocyte-Derived Dendritic Cells
Source: PLoS One. 2015 Dec 15;10(12):e0144397. doi: 10.1371/journal.pone.0144397 (PMC4684396; doi:10.1371/journal.pone.0144397)

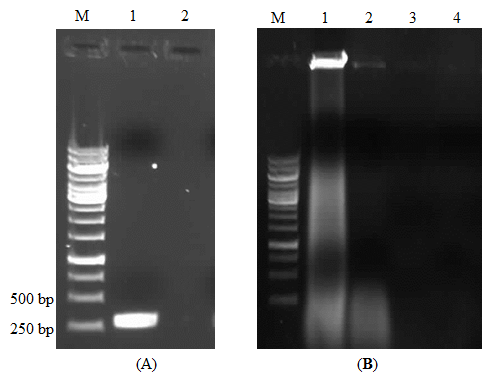

Supplement: S1 Fig — (A) Gene E was amplified from PhiX174 RFI DNA using gene specific primers. Lanes; 1, represent gene E (273 bp); 2, negative control; M, represent 1 kb marker (#SM0313, ThermoScientific, United States). (B) BGs were analysed for contamination of genomic DNA. To end this, samples were taken after induction at various time points. 1 ml of bacterial culture was centrifuged and subsequently, supernatant and pellet was analysed in 0.5% agarose gel electrophoresis. Lanes; 1, represent 0 hr culture showing genomic DNA at the well; 2, supernatant of bacterial culture post 1 hr induction showing degradation of genomic DNA; 3, pellet of bacterial culture post 4 hr induction free of genomic DNA; 4, supernatant of bacterial culture post 4 hr induction showing complete inactivation of genomic DNA; M, represent 1 kb marker. (TIF) [file pone.0144397.s001.tif]

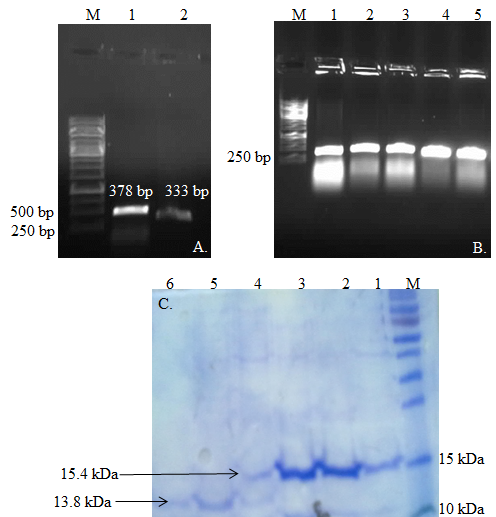

Supplement: S2 Fig — (A) GM-CSF (lane 1) and IL-4 (lane 2) were amplified using gene specific primers from bovine cDNA. Lane M represent 1 kb marker (#SM0313, ThermoScientific, United States). (B) Confirmation of GM-CSF or IL-4 gene in pET28a vector by colony PCR. Colony PCR showing amplification of 378 bp of GM-CSF (lanes 1–3) and 333 bp of IL-4 (lanes 4–5). (C) SDS–PAGE analysis of purified products of GM-CSF and IL-4. GM-CSF or IL-4 recombinant plasmid was transformed into E. coli BL21 (DE3) host strain for expression. The expressed proteins were purified by Ni-NTA cartridge as described in material methods. Lanes; 1–4, represent GM-CSF; 5–6, represent IL-4; M, molecular weight marker (#PG500-0500PI, ThermoScientific, US). (TIF) [file pone.0144397.s002.tif]
